# X‐Ray Markers for Thin Film Implants

**DOI:** 10.1002/adhm.202200739

**Published:** 2022-08-07

**Authors:** Ben J. Woodington, Lawrence Coles, Amy E. Rochford, Paul Freeman, Stephen Sawiak, Stephen J. K. O'Neill, Oren A. Scherman, Damiano G. Barone, Christopher M. Proctor, George G. Malliaras

**Affiliations:** ^1^ Electrical Engineering Division Department of Engineering University of Cambridge Cambridge CB3 0FA UK; ^2^ Department of Veterinary Medicine University of Cambridge Cambridge CB3 0ES UK; ^3^ Department of Clinical Neurosciences University of Cambridge Cambridge CB2 0QQ UK; ^4^ Melville Laboratory for Polymer Synthesis Yusuf Hamied Department of Chemistry University of Cambridge Cambridge CB2 1EW UK

**Keywords:** bioelectronics, imaging, materials, neuroscience, X‐ray

## Abstract

Implantable electronic medical devices are used in functional mapping of the brain before surgery and to deliver neuromodulation for the treatment of neurological and neuropsychiatric disorders. Their electrode arrays are assembled by hand, and this leads to bulky form factors with limited flexibility and low electrode counts. Thin film implants, made using microfabrication techniques, are emerging as an attractive alternative, as they offer dramatically improved conformability and enable high density recording and stimulation. A major limitation of these devices, however, is that they are invisible to fluoroscopy, the most common method used to monitor the insertion of implantable electrodes. Here, the development of mechanically flexible X‐ray markers using bismuth‐ and barium‐infused elastomers is reported. Their X‐ray attenuation properties in human cadavers are explored and it is shown that they are biocompatible in cell cultures. It is further shown that they do not distort magnetic resonance imaging images and their integration with thin film implants is demonstrated. This work removes a key barrier for the adoption of thin film implants in brain mapping and in neuromodulation.

## Graphical abstract

The development of flexible X‐ray markers using bismuth and barium‐infused elastomers and their integration with thin‐film bioelectronic implants is presented. This work removes a key barrier for the adoption of thin film implants in brain mapping and in neuromodulation.

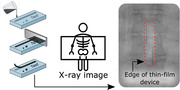

## 1 Introduction

Neuromodulation represents a new treatment frontier, where neurological function is replaced or restored with the help of electrical current stimulation from implantable electrodes. The clinical application of these devices has already revolutionized the care of many patients. Deep Brain Stimulators for Parkinson's disease, Vagus Nerve Stimulators for epilepsy, Spinal Cord Stimulators for refractory neuropathic pain, Cochlear Implants for hearing disorders are just some examples of these revolutionary technologies. Implantable electrodes are also used to map the brain to localize epileptogenic zones and guide tumor resection. Current implants are manufactured by hand, using technology that dates from the watchmaking industry. Electrodes are cut from metal tubes or metal foils, connected to metal wires, and embedded in elastomers, leading to devices that are bulky and offer limited mechanical flexibility. Advances in bioelectronics have led to the development of thin film implants, currently explored in the recording and stimulation of the brain,^[^ ^1^, ^2^, ^3^ ^]^ spinal cord,^[^ ^4^, ^5^ ^]^ and peripheral nervous system.^[^ ^6^, ^7^ ^]^ These emerging devices herald a major change in the manufacturing paradigm: They are made using microfabrication techniques developed for the electronic materials industry, which enable batch fabrication of implants with exceptional dimensional control and reproducibility. Moreover, the use of thin film fabrication techniques delivers implants with exceptional flexibility and even stretchability,^[^ ^3^ ^]^ leading to decreased glial scarring,^[^ ^8^ ^]^ and conformal contact to, e.g., the curvilinear surface of the brain's cortex.^[^ ^1^ ^]^ An additional advantage of microfabrication is that it enables exceptionally high electrode densities, which translates to precise and specific interfacing with tissues.^[^ ^9^ ^]^ Finally, thin electrodes offer intrinsic magnetic resonance imaging (MRI) compatibility,^[^ ^10^, ^11^, ^12^ ^]^ a desired feature for modern implantable devices. As a result, thin film implants have the potential to expand the use of neuromodulation and brain mapping technology by allowing more efficient interfacing with the nervous system, delivered through safer, minimally invasive surgeries.

An important clinical consideration is the ability to visualize the device during the implantation procedure. A technique which is widely employed for this purpose is X‐ray imaging, specifically computerized tommgraphy (CT) scanning and fluoroscopy.^[^ ^13^ ^]^ CT scanning is a technique widely used in neurology that can also be used to localize cortical electrodes, such as electrocoticography (ECoG) devices.^[^ ^14^ ^]^ Fluoroscopy is a particular type of live X‐ray imaging that can produce moving images by transmission of X‐rays through the body. This allows a clinician to move and manipulate the implant to ensure that it is located appropriately before testing its therapeutic potential.^[^ ^15^ ^]^

Fluoroscopy has emerged as the imaging tool of choice during the implantation and monitoring of devices on the brain,^[^ ^15^ ^]^ and the spinal cord^[^ ^16^, ^17^ ^]^ especially when restrictive or minimally invasive surgery is utilized. Current neural implants are naturally compatible with fluoroscopy, as the bulk metal electrodes attenuate X‐rays to sufficient degree to be easily visible against the contrasting tissue background. Thin film electrodes, however, do not provide enough attenuation to be visible in fluoroscopy.^[^ ^5^ ^]^ Replacing long established and standardized imaging equipment is not an appropriate or feasible strategy, so manufacturers of thin‐film implants need to consider how exactly these devices will be imaged once inside the body, using existing imaging techniques. In this work, we explore the use of bismuth‐ and barium‐infused silicones to fabricate markers for thin film implants that can be imaged using standard X‐ray fluoroscopy. We find that incorporation of these silicone‐based markers allows for devices to be visualized on the brain and spinal cord, without sacrificing the inherent flexibility of the thin film implants, the very characteristic which makes them preferable to conventional technologies. We also explore the biological safety of these markers and demonstrate MRI compatibility.

## 2 Results

### 2.1 Marker Fabrication

**Figure 1:**
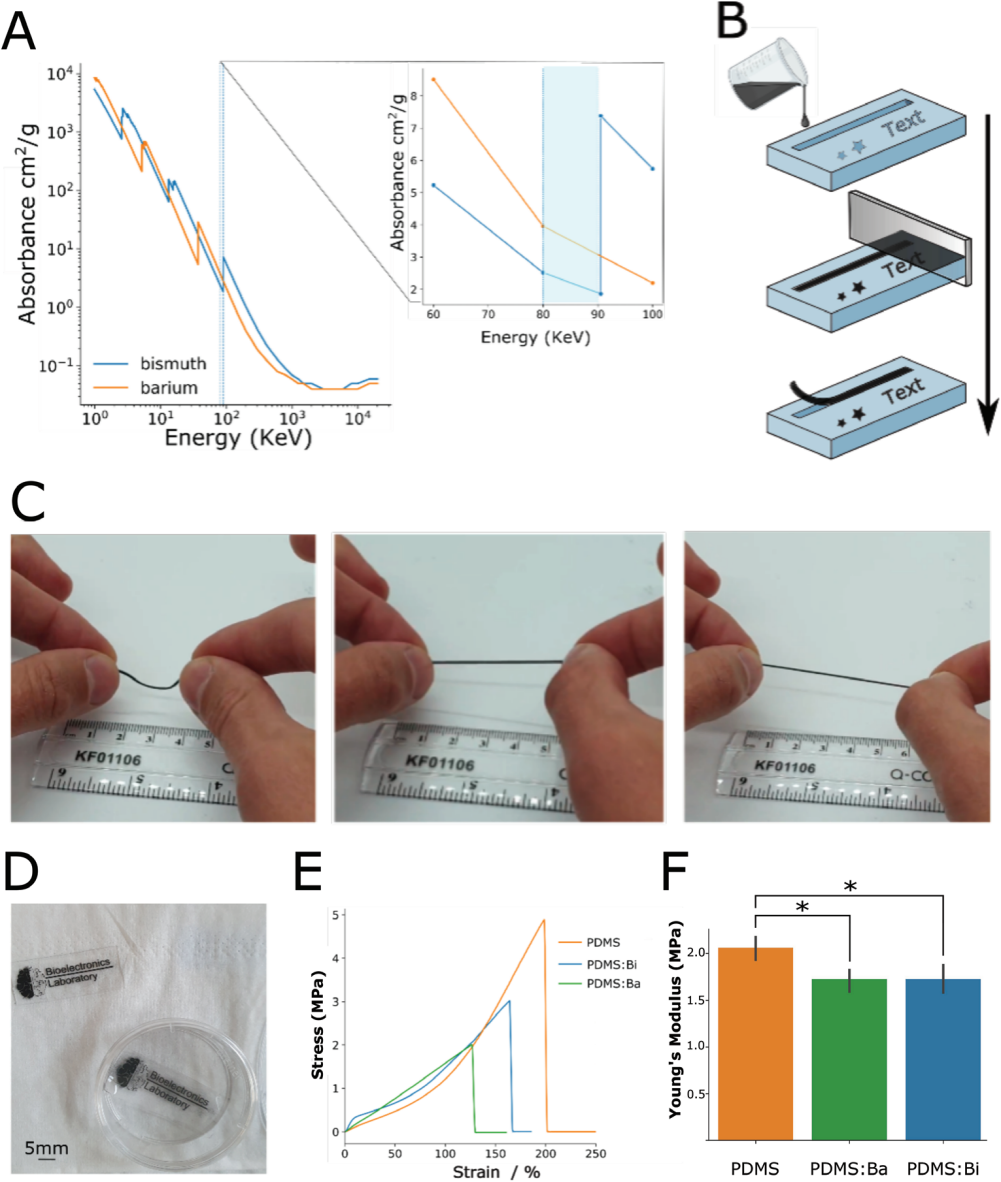
Marker fabrication. A) the relative absorbances of bismuth and barium metals, highlighted in blue and inset is the operating range of modern fluoroscopy imaging devices, absorbance data were taken from the United States Physical Measurement Laboratory (Reproduced with permission of the National Institute of Standards Agency), the full data can be viewed in Figure S1 (Supporting Information). B) Soft molding of bismuth‐ and barium‐infused silicone markers in 3D‐printed molds. C) A free‐standing marker made of a 1:2 PDMS:Bi composite shown to retain the elastic characteristics of silicone, a video of this is provided as Movie V1 (Supporting Information). D) PDMS films with radio‐opaque text and images made from a 1:2 PDMS:Bi composite. E) A stress versus strain graph comparing the elongation of a PDMS control against PDMS:Bi (1:2) and PDMS:Ba (1:1) (*n* = 1 presented for each material with *n* = 3 presented in Figure S3, Supporting Information). F) A graph plotting Young's modulus for PDMS, PDMS:Ba, and PDMS:Bi (*n* = 3 each material, results presented as mean ± SD). * *p* > 0.05%, two‐tailed *t*‐test.

Bismuth (Bi) and barium (Ba) are widely known for their strong X‐ray attenuating and shielding characteristics and their relative ease of processing.^[^ ^18^, ^19^ ^]^ They offer high absorbance in the range of photon energies used in modern fluoroscopy imaging devices (**Figure** **1**A), and have previously been used to endow X‐ray opacity in surgical tools such as gauzes,^[^ ^20^ ^]^ surgical meshes,^[^ ^21^ ^]^ and polymer tubing.^[^ ^22^ ^]^ They are, therefore, obvious choices for the development of X‐ray markers for thin film devices. We chose polydimethylsiloxane (PDMS) as a matrix to disperse these materials. PDMS is used in implantable devices, offers good flexibility and stretchability, and is compatible with a broad range of microfabrication techniques. Composites containing 1:1 and 1:2 weight ratios of PDMS to Bi were prepared by adding Bi powder into a PDMS mixture before curing (see the Experimental Section). Increasing the Bi concentration beyond the 1:2 weight ratio made it more difficult for the PDMS to fully cure. For the PDMS:Ba markers we used barium sulfate powder, as Ba is highly reactive. Weight ratios of 1:0.8 and 1:1 PDMS to BaSO_4_ powder were made, with the 1:1 ratio being the maximum concentration of powder that allowed PDMS to fully cure.

Markers were fabricated by mixing the components, pouring the mixture into 3D‐printed molds and removing excess material by doctor‐blading (Figure 1B). Upon curing, individual markers could be removed from the mold and handled as free‐standing objects. The marker shown in Figure 1C has a thickness around 700 µm and retains the flexibility and elasticity of PDMS. Markers were also incorporated onto PDMS films. For this, a two‐level mold was used, where the first level contained the marker mixture, while the second level contained pure PDMS. Figure 1D shows free standing films with markers in the shape of text and images. Both free‐standing markers and markers in PDMS films could be integrated with devices, as discussed below. Finally, Figure 1E shows tensile testing data from the PDMS:Bi (1:2) and PDMS:Ba (1:1) composites as well as from pure PDMS. Three repeats were made for each material using a 1 mm thick puck cut into a dumbbell shape. The mean strain (%) applied to each material before failure is reported for bismuth, barium, and the control sample was 158.0 (s.d 5.2), 122.0 (s.d 7.0), and 192.7 (s.d 6.8), respectively. Young's Modulus (YM) is also reported, shown in Figure 1F. The PDMS control had a measured YM of 2.06 MPa (s.d 0.11 MPa). The bismuth and barium composites had measured YM values of 1.7 MPa (s.d 0.12 MPa) and 1.7 MPa (s.d 0.15 MPa), respectively. The complete set of data are shown in Figure S3 (Supporting Information). Both bismuth and barium composites show negligible, but significant, decrease in Young's modulus when compared against PDMS control (*t*‐test comparisons reported as *p* = 0.026 and *p* = 0.037, respectively). However, the material compliance is not modified considerably by addition of either material. They also show significant elongation, with the PDMS:Bi composite outperforming the PDMS:Ba material. This was expected due to the higher volume of barium sulfate compared to that of bismuth.

### 2.2 Marker Calibration

**Figure 2:**
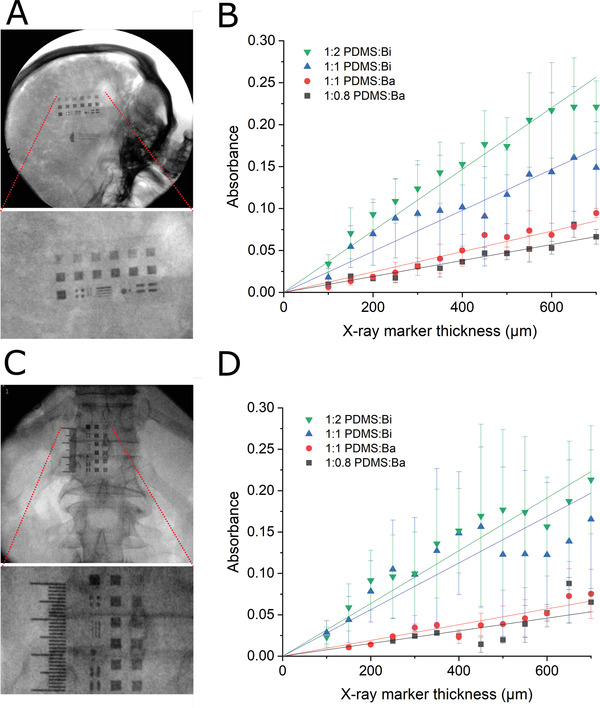
Marker calibration. A) Fluoroscopy image of a mold with PDMS:Bi markers located on the head of a human cadaver, over the brain. The ruler in the image is made with PDMS:Bi markers and shows a millimeter scale. B) Corresponding absorbance as a function of marker thickness for different compositions of the PDMS:Bi an PDMS:Ba markers. The lines are fits to the Beer–Lambert law (*n* = 3 results presented as mean ± SD). C) Fluoroscopy image of a mold with PDMS:Bi markers located on the back of a human cadaver, over the spinal cord. The ruler in the image is made with PDMS:Bi markers and shows a millimetre scale. D) Corresponding absorbance as a function of marker thickness for different compositions of the PDMS:Bi an PDMS:Ba markers. The lines are fits to the Beer–Lambert law (*n* = 3 results presented as mean ± SD).

The efficacy of markers with different compositions and thickness was investigated in human cadavers. 3D printed molds that allowed the definition of markers with 3 × 3 mm^2^ area and thickness increasing from 100 to 700 µm in steps of 150 µm were constructed (**Figure** **2**A). The molds also contained markers in the shape of smaller squares, lines, and circles with a thickness of 500 µm. The smallest feature used in fluoroscopy testing was a square with an area of 500 × 500 µm^2^. In addition, molds showing a millimeter scale were developed with 1 mm thick markers made from the 1:2 PDMS:Bi composite (Figure 2A). Figure S6 (Supporting Information) also shows the same marker thicknesses imaged using a static X‐ray machine. Here, marker dimensions were reduced to 50 and 100 µm^2^ depth at a range of sub‐500 µm^2^ dimensions, however image resolution starts to limit visibility of the marker for all materials used. For fluoroscopy cadaveric testing the two molds were placed on the head, over the brain (Figure 2A) and on the back, over the spinal cord (Figure 2C) of human cadavers and imaged using a fluoroscope. The absorbance was calculated as the logarithm of the averaged grayscale values across the marker area divided by the averaged grayscale values across a 3 × 3 mm^2^ area directly above the marker. This figure‐of‐merit ensured that attenuation from a marker is considered in the context of the background attenuation from the mold and the cadaveric anatomy beneath. The data in Figure 2B,D show increasing X‐ray attenuation with marker thickness. The lines are fits to the Beer–Lambert law. It should be noted that values of absorbance show higher variability on the spinal cord than on the brain. This is due to variations in tissue density caused by the presence of air in the lungs.

The data also show that for a given marker thickness, the higher the loading with bismuth or barium, the higher the attenuation. Finally, bismuth‐based markers show higher attenuation than barium‐based ones, which is expected as the metal in the latter is in a salt form, hence less concentrated. The marker comparison shows that the strongest attenuation is obtained with the 1:2 PDMS:Bi composite (>0.2 absorbance in both the skull and spinal experiments when using a 700 µm marker). Features as small as 500 × 500 µm^2^ made from this composite are clearly visible, as a 500 µm thick marker provides ≈30% contrast with surrounding tissue on the spinal cord and on the brain. This figure drops to ≈5–10% for a 100 µm thick marker, a contrast that still allows the marker to be visualized.

### 2.3 Biological Safety

**Figure 3:**
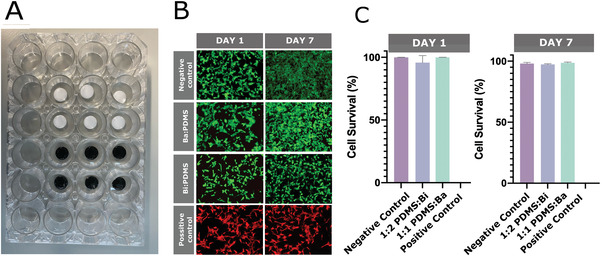
Cell viability assay. A) The well plate arrangement showing the glass discs coated with the 1:2 PDMS:Bi and the 1:1 PDMS:Ba (white) composites. B) Representative images of SH‐SY5Y from the live/dead assay on different wells at day 1 and day 7 timepoints. The live and dead cells exhibited in green and red fluorescence, respectively. Dead cells in the negative control were pretreated with 4% PFA for 30 min. C) Day 1 and 7 cell survival percentages of SY‐SH5 cells on PDMS:Bi and PDMS:Ba (*n* = 3 results presented as mean ± SD).

Although both bismuth and barium sulfate have been used in approved medical devices,^[^ ^23^, ^24^ ^]^ the new formulations with PDMS merit some biocompatibility testing. A cell viability assay with the SH‐SY5Y human neuroblastoma line was carried out to investigate the cytotoxicity of the two composites. Cylindrical glass discs coated with the 1:2 PDMS:Bi and 1:1 PDMS:Ba composites were placed into individual cell culture wells within a 24 well plate (**Figure** **3**A). Tissue culture plastic was used as a positive control representing normal growth of SH‐SY5Y cells, while for the negative control, 4% paraformaldehyde was used to kill cells grown on tissue culture plastic. A live/dead assay was performed using calcein‐AM (live) and propidium iodide (dead) stains. Fluorescence images obtained after 1 and after 7 days in culture are shown in Figure 3B. The results were analyzed, showing good cell survival on both composites, for both time points (Figure 3C), thereby confirming the lack of cytotoxicity of the two composites.

### 2.4 MRI Compatibility

**Figure 4:**
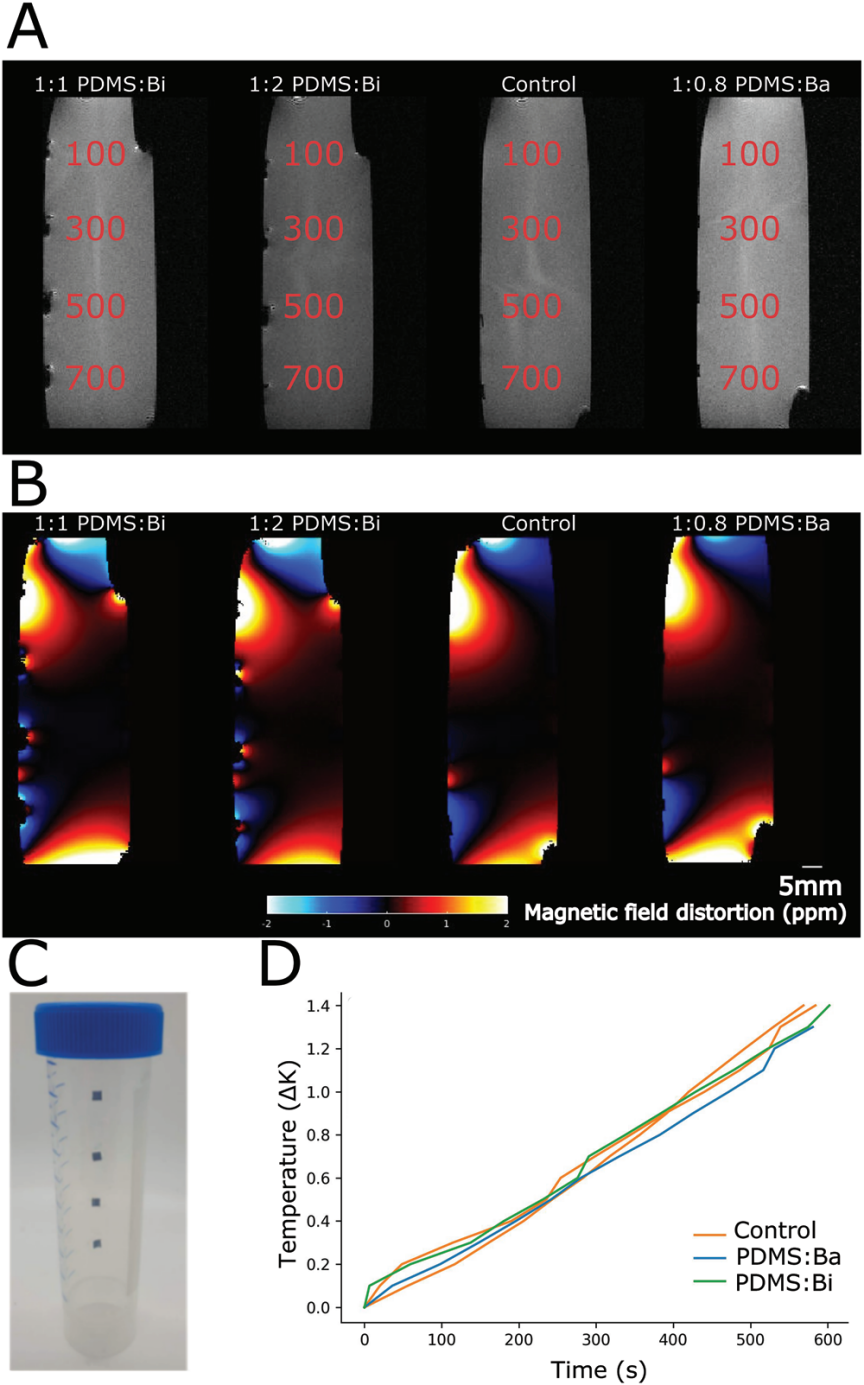
MRI compatibility. A) A MRI image comparing PDMS:Bi and PDMS:Ba markers. B) The same samples analyzed for magnetic field distortions. C) A falcon tube containing markers of different thickness is filled with water and degassed. D) Plotting the temperature change through time of the bismuth and barium PDMS composite material against a PDMS control. All samples heat at a similar rate, suggesting that only system heating effects are observed.

MRI imaging is ubiquitous in modern healthcare and even more so in neurology. There are many occasions in which patients undergoing neuromodulation treatment will need an MRI scan. Thick metal electrodes can affect the produced image quality, to the point where the image is distorted to such an extent that it renders the MRI scan useless. Thin film implants show enhanced MRI compatibility^[^ ^11^ ^]^ but it is important to screen any newly developed materials to ensure that they do not produce significant field distortions. Three tubes with markers made of PDMS:Bi (1:1 and 1:2) and PDMS:Ba (1:0.8) composites were prepared. Each tube contained four free‐standing markers with an area of 3 × 3 mm^2^ and thickness of 100, 300, 500, and 700 µm (**Figure** **4**A). A fourth tube with four pieces of plain PDMS was used as a control. The tubes were imaged with an 9.4 T MRI scanner. Figure 4A shows MRI images, with the markers placed on the left side of the tube in each scan. The markers are clearly visible in these images, and only minimal image distortions are seen near their corners. The same is true in Figure 4B that shows the calculated field distortions. Only small distortions, of the order of 1 ppm, are seen near the edges of the markers. It should be noted that the large distortions at the ends of the tubes are likely due to materials interfaces in the tube. The data suggest that the developed markers are compatible with use in an MRI scanner. Figure 4C shows the falcon tubes and markers fabricated for this testing.

Finally, a known phenomenon within MRI imaging is the possibility of heating induced by the strong RF‐fields. The highest metal loading materials PDMS:Bi 1:2 and PDMS:Ba 1:1 at the thickest level (700 µm) were tested alongside controls, using an aggressive scanning regime to assess heating effects (Figure 4D). Samples were scanned for 10 min, within this time heating effects between 1.2 and 1.4 K were observed for both the control samples and the barium or bismuth test samples. No significant difference between the samples could be seen.

### 2.5 Incorporation in Devices

**Figure 5:**
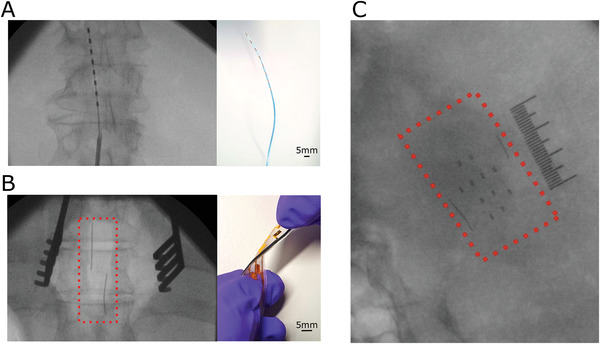
Use of markers in thin film devices. Images of a conventional percutaneous electrode array, a thin film spinal cord paddle electrode and a thin film electrocorticography array. A) Fluoroscopy image of percutaneous array and the associated device where the insertion needle and the platinum electrodes are clearly visible. B) Fluoroscopy image of the thin film paddle electrode with 500 µm PDMS:Bi markers used for navigation, inset shows the associated thin film device being twisted. C) Fluoroscopy image of a thin film cortical implant with 500 µm thick 1:2 PDMS:Bi markers at the edges and in registry with the electrode layout, next to a ruler that was also fabricated with the 1:2 PDMS:Bi composite.

A conventional percutaneous spinal cord stimulator (**Figure** **5**A) was imaged as a reference, known to be satisfactory to clinicians when positioning devices. This device comprises PtIr electrodes, which have a thickness in the range of hundreds of µm. The X‐ray image was obtained during placement of the device on the spinal cord of a human cadaver. The eight PtIr electrodes, the wires that connect them to the stimulator, and the percutaneous needle that is used to implant the device on the spinal cord are clearly visible. Figure 5B shows the same data for a paddle electrode placed in the spinal cord of a human cadaver. The device consisted of a 4 µm thick parylene‐C film with embedded Au electrodes attached to an 80 µm thick PDMS film to facilitate surgical handling. The electrodes were patterned with photolithography and have a thickness of 100 nm, as described in a previous publication.^[^ ^5^ ^]^ Free‐standing markers made of the 1:2 PDMS:Bi composite were incorporated by placing them into the uncured layers of PDMS during device fabrication then allowing them to cure. The markers, which had a thickness of 500 µm, remain robustly affixed to the device even after bending (Figure 5B). Neither the Au electrodes, nor the interconnects or the parylene C/PDMS substrate are visible in the X‐ray image. In contrast, the two markers allow the edges of the implant to be visualized. The same holds true for a cortical electrode array, placed on the brain of a human cadaver (Figure 5C). In this implant, markers were also placed in registry with the electrode layout, to allow more complete visualization of the device. A second device image with an alternate marker layout is provided as Figure S3 (Supporting Information). These results demonstrate the successful use of markers in thin film implants on the brain and the on spinal cord.

## 3 Discussion

The ability to image thin film implants using fluoroscopy is going to be essential for their success as clinical neuromodulation and brain mapping tools. In this work, we show that thin film implants can be made X‐ray opaque using markers made of PDMS infused with bismuth and barium. The markers retain the compliance characteristics of PDMS with similar Young's moduli, however the composite materials show decreased performance in tensile testing, revealed by smaller elongation to break. The materials can be produced as free‐standing structures or integrated with a PDMS film. Given the widespread use of PDMS in microfluidics, several patterning techniques have been developed for this material.^[^ ^5^, ^25^, ^26^ ^]^ These techniques can be easily adapted to incorporate markers in a variety of process flows and device formats.

We show calibration curves that relate the composition and thickness of these materials to the attenuation of X‐rays. These can inform the design of markers for specific applications. We demonstrate that composites with a similar thickness and loading capacity present differently when imaging the head and spinal cord. There is often more tissue to penetrate in the thorax as well as air in the lungs, which can lead to difficulty in imaging. The skull and scalp meanwhile are relatively thin, more homogenous, and exhibit less variability between patients. Though not demonstrated here, the peripheral nerves would likely have an even thinner layer of attenuating tissue so they would be easier to image, potentially allowing thinner X‐ray opaque markers to be integrated into peripheral nerve interfaces. We offer evidence that the developed markers are not cytotoxic in neuronal‐like environments. Any use of these materials, however, in medical devices would require extensive in vivo characterization in preclinical animal studies, and experiments to investigate possible leaching of the metals. These studies would be performed in the context of a particular device. We further investigate the MRI compatibility of these markers. MRI compatibility is becoming an essential feature for neuromodulation devices, as it ensures that implanted devices do not need to be explanted, adding cost and complexity to patients and their clinicians.^[^ ^17^, ^18^ ^]^ Although thin film‐based implants show improved MRI compatibility compared to conventional ones, any additional component, including any markers, should be carefully evaluated. We show that the developed markers have minimal impact on MRI images and therefore maintain the MRI compatibility of thin film implants. We also show minimal heating of the samples, comparable to the control samples, suggesting any observed heating is dominated by system heating effects In summary, we demonstrate a marker technology that allows thin film implants to be visualized with fluoroscopy, thereby removing a significant a barrier for the adoption of these devices in neuromodulation and brain mapping.

## 4 Experimental Section

### Fabrication

To create the markers, fine bismuth or barium sulfate powder (Sigma‐Aldich) was mixed into a two‐part cure PDMS silicone (Sylgard 184, Dow). A 1:10 ratio of curing agent to silicone base was used. The powder was added at the desired ratio, and the mixture was combined in a Speedmixer for 90 s at 1800 rpm. This mixture was degassed under vacuum, poured into a 3D printed mold allowed to cure at 50–70 °C for at least 1 h. The molds were made from a Freeprint Temp UV A1 resin (Dentax, iMakr) on a Asiga Max 3D printer. They were degassed under vacuum for at least 2 h before coated with a 2 µm parylene‐C layer. This aided in the curing and removal of the markers. The molds were filled with uncured polymer composite, which was blade coated to ensure a consistent fill. The composites were degassed and cured in a vacuum oven at 50–70 °C for 60–120 min, then removed from the mold. To create films with text and images, a two‐level mold was used. The first level contained the markers, while the second level contained a thin layer of pure PDMS that held them together after removal. The two levels were degassed and cured together in a vacuum oven at 50–70 °C for at least 1 h, then removed from the mold.

### Tensile Testing

Three materials were prepared for tensile testing, PDMS:Bi (1:2), PDMS:Ba (1:1), and a PDMS control were tested in triplicate. These samples were prepared in a petri dish to 1 mm in thickness, degassed under vacuum and cured at 65 °C for 2 h. Tensile tests were performed on a Tinius Olsen Model HK 25 kN Benchtop Tester machine equipped with a 25‐N load cell at room temperature. A typical tensile test was performed by stretching a dumbbell‐shaped specimen (following ISO4661‐1 standard) at 50 mm min^−1^ until its breakage, while measuring the force required, to obtain the stress–strain curve. The equipment used in this testing is shown in Figure S5 (Supporting Information).

### Cadaveric Investigation

The X‐ray markers were imaged on three separate fresh‐frozen human cadavers at the Evelyn Surgical Training Centre, Cambridge (UK). The markers were imaged using a Siemens Siremobil compact L fluoroscope, with the markers placed within their molds on the scalp and the back above the spine of the cadavers. The voltage of the fluoroscope was set automatically in the range of 80–90 kV by the machine.

### Biological Safety

Cylindrical glass discs coated with PDMS:Bi and PDMS:Ba were placed into individual cell culture wells within a 24 well plate (Corning). Both positive and negative controls were used for comparison. Tissue culture plastic was used as positive control representing normal growth of cells. 4% paraformaldehyde was used as a negative control as it kills the cells (0% viability). The materials in wells were plasma treated at 25 W for 60 s to make the surface hydrophilic for cell culture. The inside of the wells was kept wet from this point on with DI water. The well plates were sterilized for 30 min in 70% ethanol and rinsed with Dulbecco's phosphate‐buffered saline (DPBS). Next, each disc was coated with Corning Matrigel matrix (Cat. No. 354 234 or 354 230), defrosted on ice to avoid polymerization. 0.1 mL (200–300 µg mL^−1^) corresponding to 50 µL cm^−2^ of Corning Matrigel matrix was added to each well containing the discs. A human neuroblastoma (SH‐SY5Y) cell line (ATTC) was cultured in Dulbecco's modified Eagle's culture medium (DMEM, Sigma‐Aldrich) and was supplemented with nonheat activated 10% fetal bovine serum (FBS, Sigma‐Aldrich). When the cells reached 80% confluency, the cells were washed with Hanks' Balanced Salt Solution (Thermo Fisher) and collected by trypsinization using 0.25% Tripsin‐EDTA solution in PBS (Sigma‐Aldrich). Cells were counted by an automated cell counter (BioRad) and seeded at density of 1.5 × 10^5^ per well. Cells were grown in humidified conditions (95% humidity), at 37 °C, 5% CO_2_ for up to 7 d. A live/dead assay [LIVE/DEAD Cell Imaging Kit (488/570) ThermoFisher] was carried out to examine the viability of SH‐SY5Y cells on the composites. The Live (Calcein‐AM solution) and Dead (Propidium iodide solution) were defrosted at room temperature and 10 µL of each solution were combined to create a working solution. Equal amounts of the working solution were added to each well and incubated for 15 min at 20–25 °C. Next, SH‐SY5Y cells were imaged using an upright Echo revolve upright microscope. The fluorophores used were detected using the FITC and Texas Red filters. The imaging processing software Fiji was used. The obtained images were converted into single channel, 8 bit images. To quantify the signal in the green and red channels, the images were split and treated separately. To reduce the background noise, brightness, and contrast were adjusted appropriately. To count live and dead cells the gray‐scale images were converted into binary black and white images. Regions of interest (ROI) were set based on the average dimension of the neurons. To calculate the percentage of live cells, the number of spots detected in the green channel was divided by the total number of green and red cells in the image and the outcome was multiplied by 100%.

### MRI Imaging

Four free‐standing pieces of PDMS:Bi (1:1 or 1:2), PDMS:Ba (1:0.8) or PDMS were affixed on the inside of 50 mL falcon tubes (Fisher). The pure PDMS was used as a control sample. The tubes were filled with deionized water and sonicated for 10–20 s to remove any air bubbles trapped in the sample as these would lead to MRI artifacts. The tubes were sealed and imaged using a Bruker BioSpec 94/20 9.4T MRI scanner. Images of the tubes were acquired using the manufacturer‐provided 12 cm quadrature volume resonator. The sequence used was a dual‐echo 3D gradient echo sequence (repetition time 10 ms; echo times 2.22 ms, 5.77 ms; flip angle 6°; bandwidth 100 kHz). The field of view was 80.1 × 40.0 × 40.0 mm^3^ with a matrix of 256 × 128 × 128, yielding an isotropic resolution of 0.313 mm. Standard image reconstruction was used to visualize artefacts arising from the devices in addition to a field‐map reconstruction that scaled the phase difference between echo images to measure local frequency offsets. These vary naturally over an extended field of view and in particular at material interfaces, but rapid changes near the devices can reveal their potential to cause image distortion and other MR artefacts.

For heating studies markers were scanned with an RF‐intensive sequence (turbo spin‐echo with a train of 32 180° pulses (1.3 ms, bandwidth 2.6 kHz, separation 4.5 ms) per slice ×12 slices, phase encoding repetition time (TR) 1.8 s; repeated 42 times). The sequence ran for 10 min 4 s. In addition to any direct RF heating effects, this intensive sequence causes heating of the MRI system gradients, RF transmitter coil, and the receiver coil.

All markers (or control substrates) were scanned in a 50 mL tube of water with this sequence with an MRI‐compatible thermister (Model 1030T animal monitoring system, SA Instruments, Stony Brook, NY) taped to the tube at the level of the device (or control). This was repeated four times (control, device, device, control) to control for system heating.

### Devices

A percutaneous spinal cord stimulator (Figure 5A) (Nevro) was imaged. The spinal cord device (Figure 5B) was fabricated as previously reported.^[^ ^5^ ^]^ The ECoG device (Figure 5C) was fabricated by spin coating layers of PDMS (Sylgard 184, Dow) following the protocol outlined previously.^[^ ^5^ ^]^

### Statistical Analysis

All data are presented without preprocessing unless otherwise stated in the relevant section. No outliers have been removed from the data and all points are plotted on relevant graphs. Data are presented is mean and standard deviation (SD). Data were processed using Python 3.x (pandas, matplotlib, seaborn, and scipy) for Figures 1, 3, and 4 and MATLAB r2021a for Figure 2.

### Ethical Statement

Measurements on cadavers were performed in the Evelyn Cambridge Surgical Training Centre under Human Tissue Authority license (12603).

## Conflict of Interest

The authors declare no conflict of interest.

## Author Contributions

B.J.W. and L.C. contributed equally to this work. B.J.W. and L.C. led the design, fabrication, testing, and data analysis involved in this project, and compiled the first draft of this work. S.J.K.O. and O.A.S. provided the mechanical characterization of the composites. A.E.R. provided the cell viability testing and created the associated figures. S.S. provided all MRI data. P.F. provided additional X‐ray data. D.G.B. led the work on human cadavers. D.G.B. and C.M.P. reviewed and edited the manuscript, G.G.M. produced the final draft. G.G.M., C.M.P., and D.G.B. oversaw the project.

## Supporting information

Supporting Information

Supplemental Video 1

## Data Availability

The data that support the findings of this study are available from the corresponding author upon reasonable request.
