# Supplementary material for: X‐Ray Markers for Thin Film Implants
Source: Adv Healthc Mater. 2022 Aug 7;11(18):2200739. doi: 10.1002/adhm.202200739 (PMC11468128; doi:10.1002/adhm.202200739)
Supplement: Supplementary file 1 — Supporting Information [file ADHM-11-2200739-s002.pdf]

ADVANCED HEALTHCARE MATERIALS

Supporting Information

for *Adv. Healthcare Mater.*, DOI 10.1002/adhm.202200739

X-Ray Markers for Thin Film Implants

*Ben J. Woodington, Lawrence Coles, Amy E. Rochford, Paul Freeman, Stephen Sawiak, Stephen J. K. O'Neill, Oren A. Scherman, Damiano G. Barone, Christopher M. Proctor and George G. Malliaras**

X-ray markers for thin film implants

Supplementary Information

Ben J. Woodington[‡1], Lawrence Coles[‡1], Amy E. Rochford[1], Paul Freeman[3], Stephen Sawiak[2], Stephen J.K. O'Neil[4], Oren A. Sherman[4], Damiano G. Barone[†1,2], Christopher M. Proctor[†2], George G. Malliaras[†2]

Author information
‡Co-first authors
†Corresponding authors: gm603@cam.ac.uk (GGM); cmp81@cam.ac.uk (CMP); dgb36@cam.ac.uk (DGB)

Affiliations
1. Electrical Engineering Division, Department of Engineering, University of Cambridge, Cambridge, CB3 0FA, United Kingdom
2. Department of Clinical Neurosciences, University of Cambridge, Cambridge, CB2 0QQ, United Kingdom
3. Department of Veterinary Medicine, University of Cambridge, Cambridge, CB3 0ES, United Kingdom
4. Melville Laboratory for Polymer Synthesis, Yusuf Hamied Department of Chemistry, University of Cambridge, Cambridge, CB2 1EW, United Kingdom

Barium
$Z = 56$

HTML table format

	Energy (MeV)	μ/ϱ (cm^2/g)	μ_{en}/ϱ (cm^2/g)
	1.00000E-03	8.543E+03	8.521E+03
	1.03063E-03	7.990E+03	7.969E+03
	1.06220E-03	7.465E+03	7.445E+03
M3	1.06220E-03	8.547E+03	8.524E+03
	1.09882E-03	7.957E+03	7.935E+03
	1.13670E-03	7.404E+03	7.384E+03
M2	1.13670E-03	7.837E+03	7.815E+03
	1.21224E-03	6.861E+03	6.841E+03
	1.29280E-03	5.985E+03	5.968E+03
M1	1.29280E-03	6.255E+03	6.237E+03
	1.50000E-03	4.499E+03	4.485E+03
	2.00000E-03	2.319E+03	2.308E+03
	3.00000E-03	8.696E+02	8.622E+02
	4.00000E-03	4.246E+02	4.184E+02
	5.00000E-03	2.414E+02	2.361E+02
	5.24700E-03	2.135E+02	2.084E+02
L3	5.24700E-03	6.098E+02	5.689E+02
	5.43204E-03	5.634E+02	5.263E+02
	5.62360E-03	5.169E+02	4.835E+02
L2	5.62360E-03	7.016E+02	6.486E+02
	5.80333E-03	6.507E+02	6.025E+02
	5.98880E-03	6.013E+02	5.577E+02
L1	5.98880E-03	6.930E+02	6.412E+02
	6.00000E-03	6.898E+02	6.383E+02
	8.00000E-03	3.334E+02	3.127E+02
	1.00000E-02	1.860E+02	1.754E+02
	1.50000E-02	6.347E+01	5.972E+01
	2.00000E-02	2.938E+01	2.730E+01
	3.00000E-02	9.904E+00	8.875E+00
	3.74406E-02	5.498E+00	4.768E+00
K	3.74406E-02	2.919E+01	9.346E+00
	4.00000E-02	2.457E+01	8.842E+00
	5.00000E-02	1.379E+01	6.534E+00
	6.00000E-02	8.511E+00	4.660E+00
	8.00000E-02	3.963E+00	2.501E+00
	1.00000E-01	2.196E+00	1.470E+00
	1.50000E-01	7.828E-01	5.309E-01
	2.00000E-01	4.046E-01	2.583E-01
	3.00000E-01	1.891E-01	1.028E-01
	4.00000E-01	1.265E-01	6.125E-02
	5.00000E-01	9.923E-02	4.521E-02
	6.00000E-01	8.410E-02	3.743E-02
	8.00000E-01	6.744E-02	3.011E-02
	1.00000E+00	5.803E-02	2.657E-02
	1.25000E+00	5.058E-02	2.388E-02
	1.50000E+00	4.592E-02	2.224E-02
	2.00000E+00	4.078E-02	2.069E-02
	3.00000E+00	3.692E-02	2.044E-02
	4.00000E+00	3.598E-02	2.135E-02
	5.00000E+00	3.612E-02	2.250E-02
	6.00000E+00	3.669E-02	2.363E-02
	8.00000E+00	3.844E-02	2.566E-02
	1.00000E+01	4.042E-02	2.731E-02
	1.50000E+01	4.518E-02	3.003E-02
	2.00000E+01	4.902E-02	3.126E-02

Bismuth
$Z = 83$

HTML table format

	Energy (MeV)	μ/ϱ (cm^2/g)	μ_{en}/ϱ (cm^2/g)
	1.00000E-03	5.441E+03	5.427E+03
	1.50000E-03	2.468E+03	2.455E+03
	2.00000E-03	1.348E+03	1.336E+03
	2.57960E-03	7.724E+02	7.612E+02
M5	2.57960E-03	1.777E+03	1.731E+03
	2.63305E-03	1.850E+03	1.799E+03
	2.68760E-03	1.852E+03	1.802E+03
M4	2.68760E-03	2.576E+03	2.500E+03
	3.00000E-03	2.053E+03	1.996E+03
	3.17690E-03	1.774E+03	1.725E+03
M3	3.17690E-03	2.048E+03	1.992E+03
	3.42677E-03	1.707E+03	1.659E+03
	3.69630E-03	1.415E+03	1.378E+03
M2	3.69630E-03	1.498E+03	1.460E+03
	3.84472E-03	1.366E+03	1.329E+03
	3.99910E-03	1.243E+03	1.211E+03
M1	3.99910E-03	1.297E+03	1.264E+03
	4.00000E-03	1.296E+03	1.263E+03
	5.00000E-03	7.580E+02	7.386E+02
	6.00000E-03	4.855E+02	4.721E+02
	8.00000E-03	2.378E+02	2.295E+02
	1.00000E-02	1.360E+02	1.298E+02
	1.34186E-02	6.491E+01	6.064E+01
L3	1.34186E-02	1.560E+02	1.234E+02
	1.50000E-02	1.160E+02	9.358E+01
	1.57111E-02	1.027E+02	8.346E+01
L2	1.57111E-02	1.416E+02	1.069E+02
	1.60457E-02	1.351E+02	1.024E+02
	1.63875E-02	1.282E+02	9.769E+01
L1	1.63875E-02	1.478E+02	1.116E+02
	2.00000E-02	8.952E+01	7.060E+01
	3.00000E-02	3.152E+01	2.617E+01
	4.00000E-02	1.495E+01	1.255E+01
	5.00000E-02	8.379E+00	7.004E+00
	6.00000E-02	5.233E+00	4.320E+00
	8.00000E-02	2.522E+00	1.999E+00
	9.05259E-02	1.856E+00	1.434E+00
K	9.05259E-02	7.380E+00	2.073E+00
	1.00000E-01	5.739E+00	1.951E+00
	1.50000E-01	2.082E+00	1.071E+00
	2.00000E-01	1.033E+00	6.014E-01
	3.00000E-01	4.163E-01	2.532E-01
	4.00000E-01	2.391E-01	1.414E-01
	5.00000E-01	1.656E-01	9.411E-02
	6.00000E-01	1.277E-01	7.017E-02
	8.00000E-01	9.036E-02	4.758E-02
	1.00000E+00	7.214E-02	3.730E-02
	1.25000E+00	5.955E-02	3.039E-02
	1.50000E+00	5.285E-02	2.678E-02
	2.00000E+00	4.659E-02	2.389E-02
	3.00000E+00	4.279E-02	2.347E-02
	4.00000E+00	4.242E-02	2.475E-02
	5.00000E+00	4.317E-02	2.626E-02
	6.00000E+00	4.437E-02	2.772E-02
	8.00000E+00	4.725E-02	3.020E-02
	1.00000E+01	5.025E-02	3.213E-02
	1.50000E+01	5.721E-02	3.514E-02
	2.00000E+01	6.276E-02	3.632E-02

Figure S1: Complete x-ray absorbance data for barium and bismuth as provided by the National Institute of Standards and Technology.

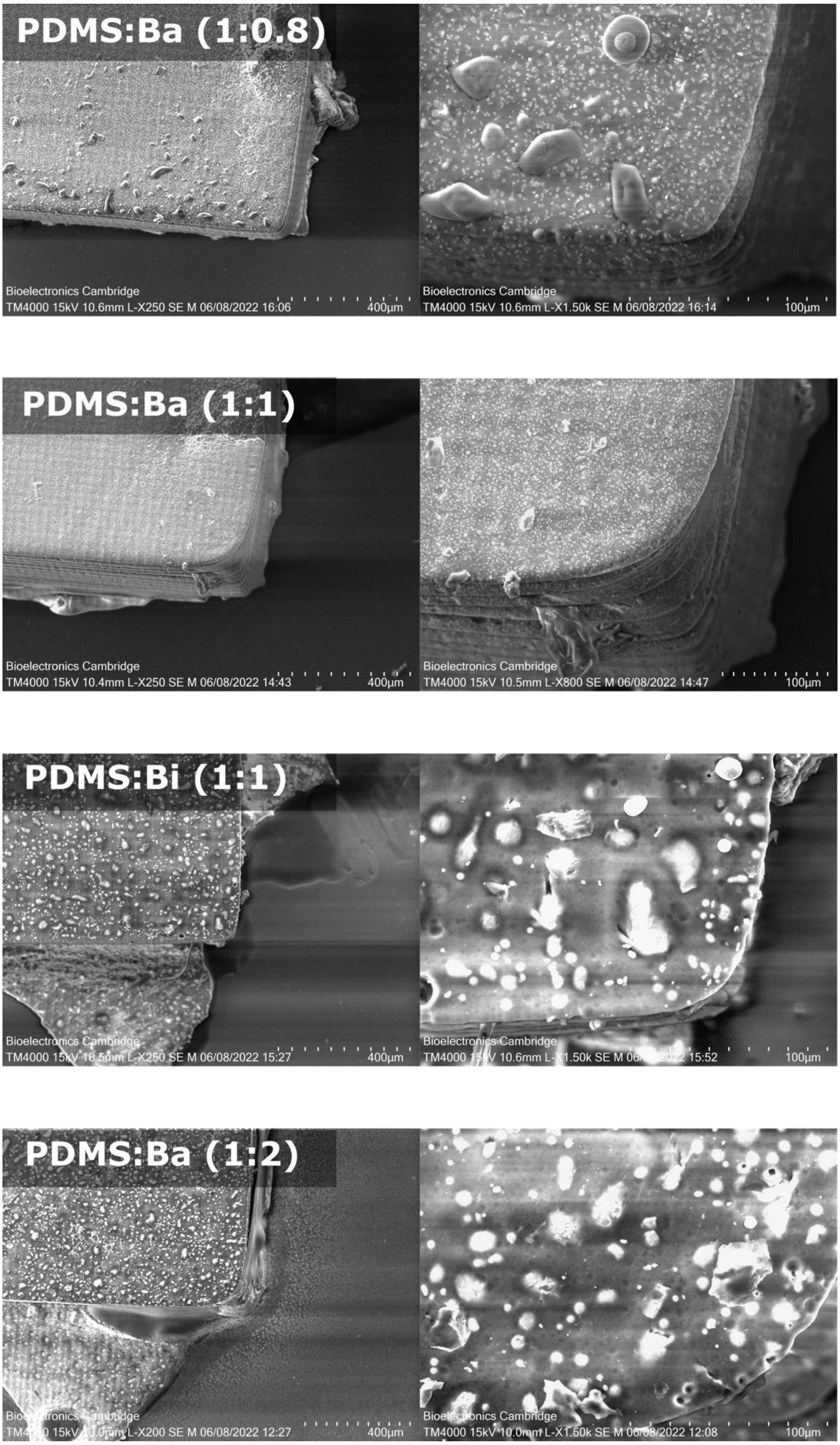

Figure S2: SEM images of PDMS:Bi and PDMS:Ba markers. Images were captured using a Hitachi TM4000Plus benchtop scanning electron microscope. A scale bar at the base of the image indicates particle size.

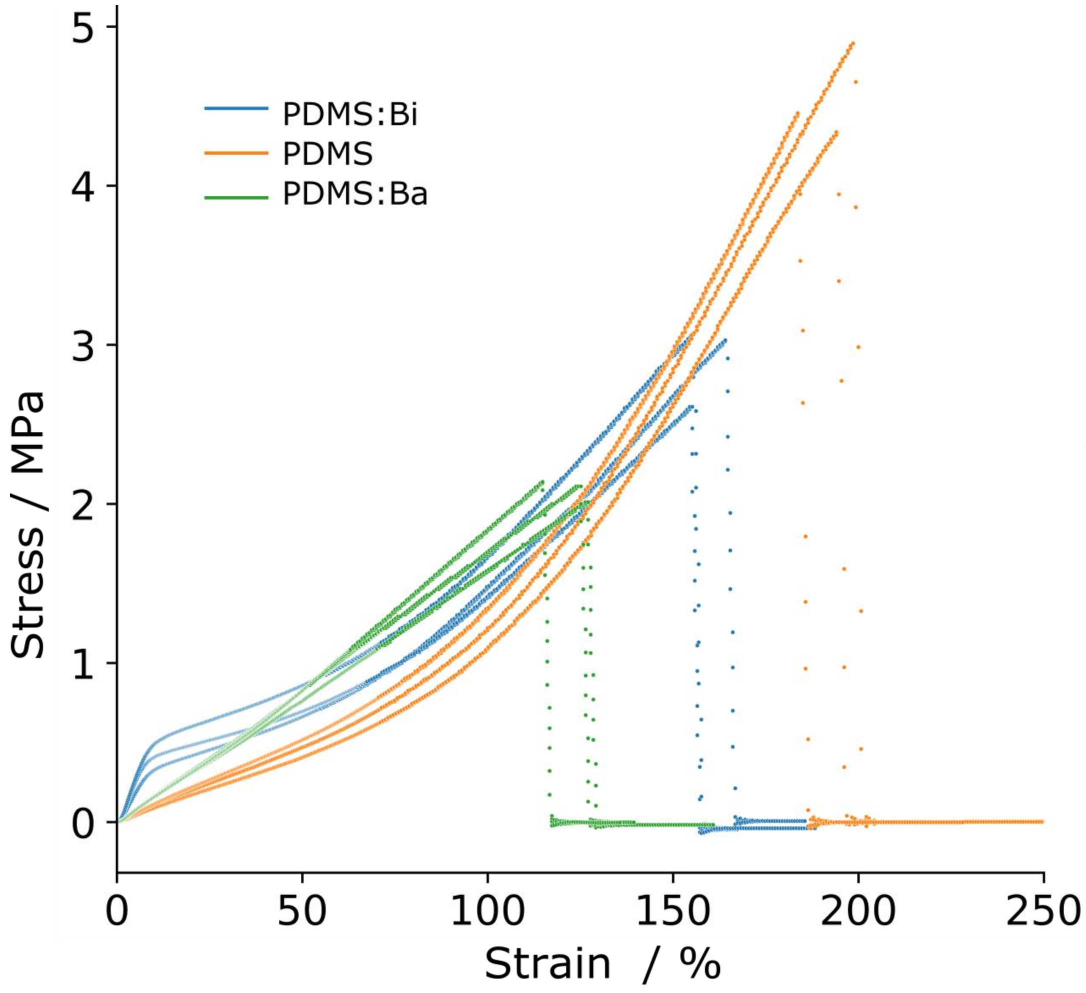

Figure S3: Three repeats for each material carried out for tensile testing.

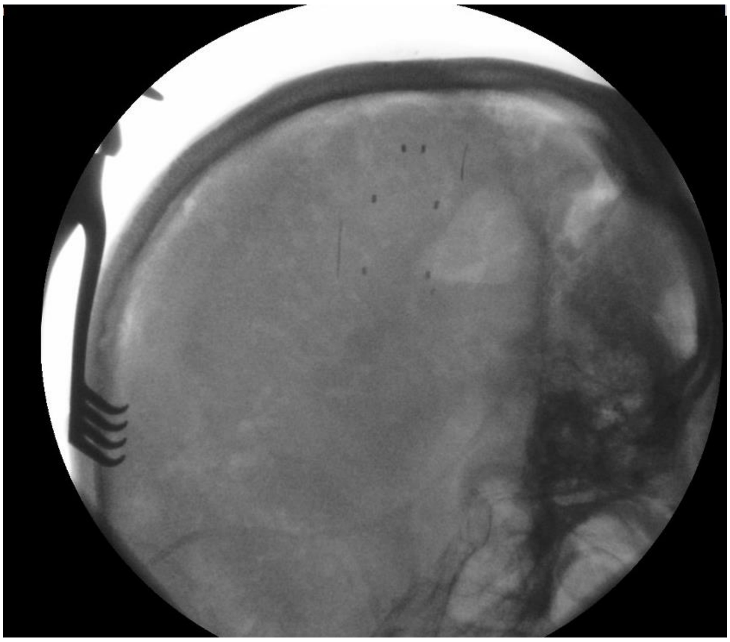

Figure S4: A fluoroscopy image of an ECoG device on the cortical surface of a human cadaver with PDMS:Bi markers used to indicate electrode position.

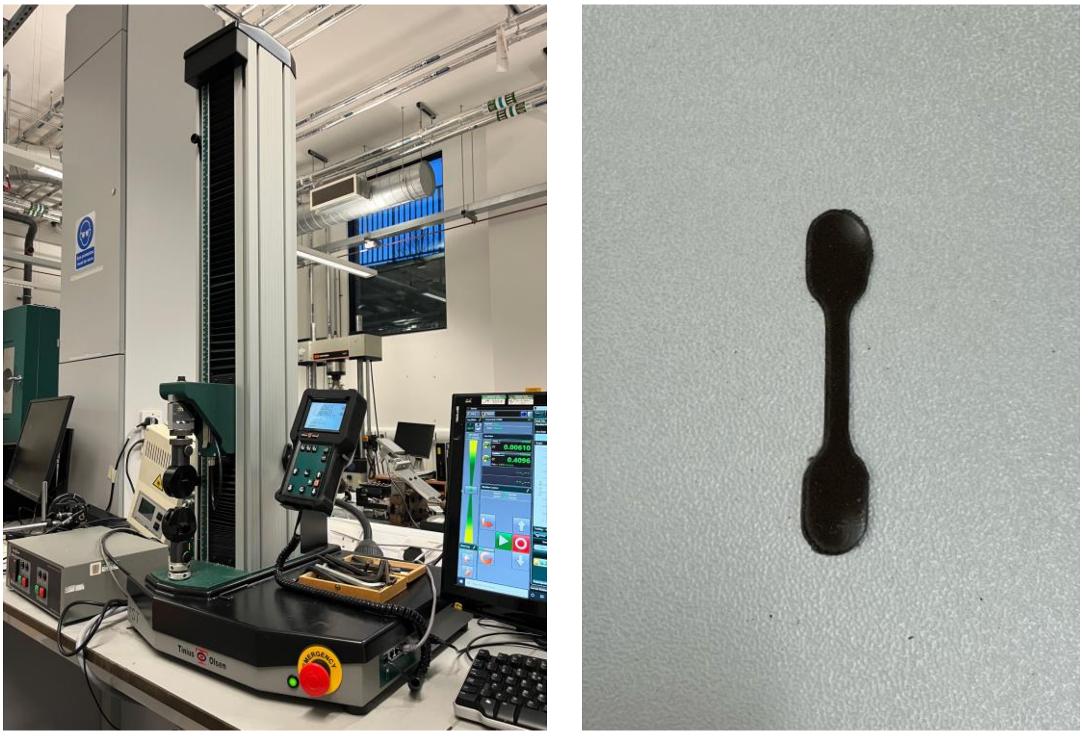

Figure S5: The equipment used to carry out the tensile testing of the PDMS-based materials (right) and an example 'dumbbell' which has been cut from a puck of PDMS:Bi at a ratio of 1:2.

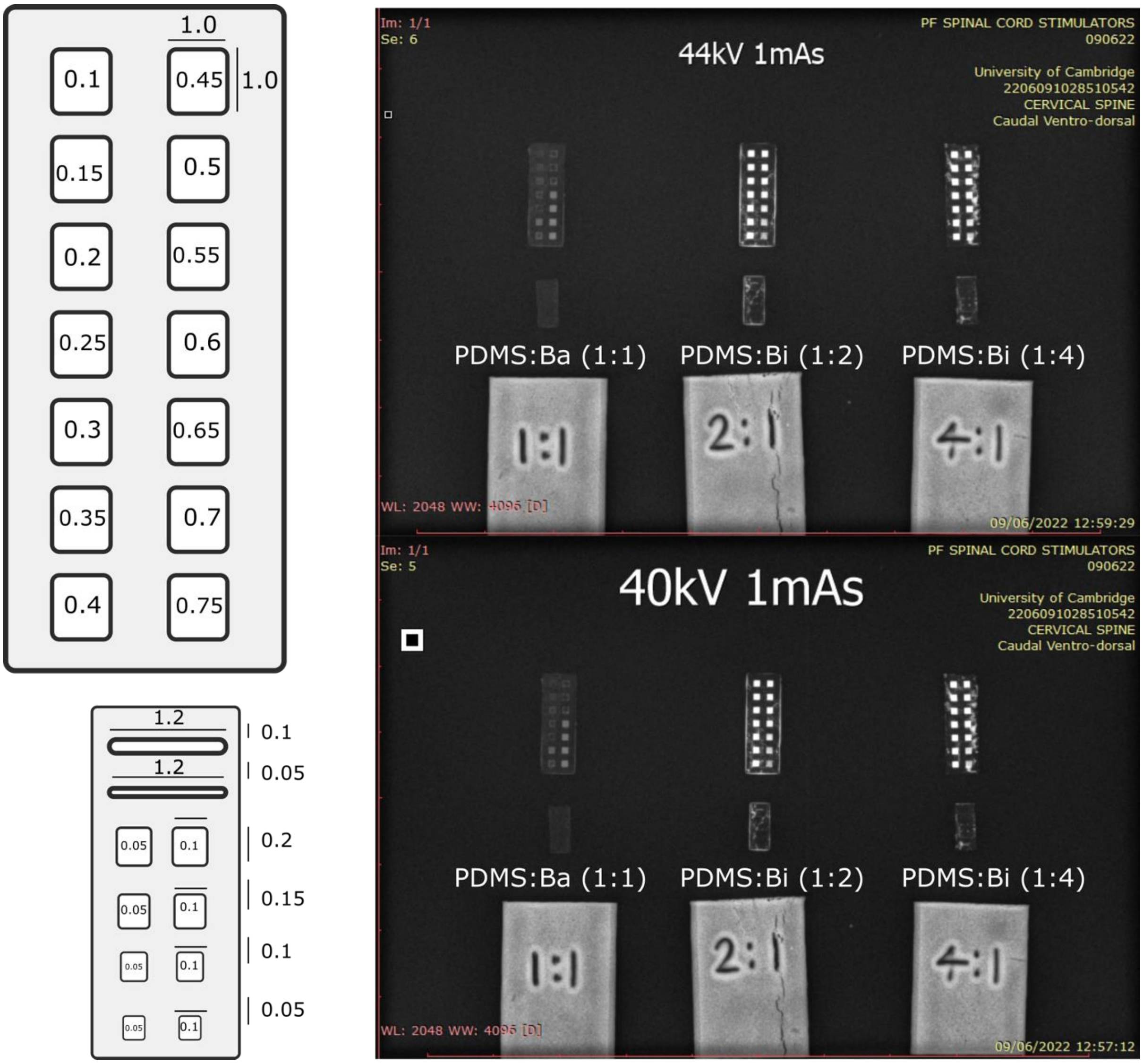

Figure S6: Three moulds filled with PDMS:Bi and PDMS:Ba material from a static x-ray image. The mould dimensions are shown to the left of the image with the depth of the moulds written inside the wells. Two repeats were performed at 40kV and 44kV.

Movie S1: An example of the material being stretched showcasing the PDMS:Bi retained elasticity at a high loading volume (1:2)
